# Increased sensitivity of the updated FluoroType MTBDR V2 with addition of IS*6110* analysis

**DOI:** 10.1128/jcm.00268-26

**Published:** 2026-06-23

**Authors:** Erik Svensson, Dorte Bek Folkvardsen, Anders Norman, Erik Michael Rasmussen, Troels Lillebaek

**Affiliations:** 1 International Reference Laboratory of Mycobacteriology, Statens Serum Institut 4326, Copenhagen, Denmark; 2 Global Health Section, Department of Public Health, University of Copenhagen 4321, Copenhagen, Denmark; The University of North Carolina at Chapel Hill School of Medicine, Chapel Hill, North Carolina, USA

**Keywords:** *Mycobacterium tuberculosis *complex, IS*6110*, FluoroType MTBDR, drug-resistant tuberculosis, moderate-complexity automated nucleic acid amplification tests, paucibacillary tuberculosis

## Abstract

Tuberculosis (TB) remains a major global health challenge, requiring rapid, reliable molecular tests to detect the *Mycobacterium tuberculosis* complex (MTBC) and resistance to the drugs that cause multidrug resistance, i.e., rifampicin and isoniazid. Although many moderate-complexity automated nucleic acid amplification tests use multiple targets to improve sensitivity, previous versions of FluoroType MTBDR (FTv2old; Bruker-Hain) relied on a single MTBC DNA target, often necessitating the use of an initial multilocus MTBC assay in the workflow to achieve comparable sensitivity. This prospective head-to-head study compared FTv2old with an updated version (FTv2new) that includes the multi-copy IS*6110* insertion sequence to improve MTBC detection. Using 1,483 clinical specimens from pulmonary and extrapulmonary sources, with culture and whole-genome sequencing (WGS) as reference standards, FTv2new showed a significant 17-percentage-point increase in overall sensitivity, reaching 87% compared with 70% for FTv2old (*P* = 0.008). The improvement was particularly notable in microscopy-negative, culture-positive samples, where the sensitivity increased from 50% to 78% (*P* = 0.008). Specificity remained high and comparable between versions at 99%. Because the assay’s resistance-conferring targets remained unchanged, the capability for resistance profiling did not improve significantly. These findings indicate that inclusion of the IS*6110* target closes the performance gap of the FluoroType platform, eliminating the need for reflex testing and streamlining laboratory workflows for high-throughput tuberculosis screening.

**IMPORTANCE:**

Tuberculosis remains a major global health threat, and rapid, accurate diagnosis is essential for disease control. This study evaluates an updated molecular assay for detecting the *Mycobacterium tuberculosis* complex (MTBC). Earlier versions of this assay relied on a single genetic target and were less sensitive than competing methods. The inclusion of a multi-copy target significantly improves MTBC detection, particularly in specimens with low bacterial loads that are frequently microscopy-negative. This improvement is clinically relevant, enabling faster, more reliable diagnosis while eliminating the need for additional, time-consuming reflex testing, thereby streamlining laboratory workflows and supporting timely initiation of appropriate treatment, thereby supporting global efforts to eliminate TB.

## INTRODUCTION

Tuberculosis (TB), caused by *Mycobacterium tuberculosis* complex (MTBC) members, remains a major global health concern. According to the World Health Organization (WHO) Global TB Report 2025, an estimated 10.8 million individuals developed TB in 2023, resulting in 1.25 million deaths (1). Rifampicin (RIF) and isoniazid (INH) are the backbone drugs of TB treatment; multidrug-resistant TB (MDR-TB) is, therefore, defined as resistance to both RIF and INH, while RIF resistance alone (RR-TB) is used as a proxy for MDR-TB.

The treatment of MDR/RR-TB is substantially more expensive than the standard TB therapy and remains a major global challenge, with an estimated 410,000 MDR/RR-TB cases annually, of whom only two in five are initiated on an appropriate treatment (1). In addition, there is a high global burden of INH-resistant, RIF-susceptible TB, estimated to account for 13.1% of new cases, and INH resistance frequently remains undiagnosed when assays are designed to detect RIF resistance only (2).

To address these diagnostic gaps, the WHO Consolidated Guidelines and Operational Handbook on Tuberculosis recommend using rapid nucleic acid amplification tests (NAATs) as the initial diagnostic test for all individuals with signs and symptoms of TB (2, 3). Under the current WHO nomenclature, these platforms are categorized by complexity. Low-complexity automated NAATs (LC-aNAATs), such as Xpert MTB/RIF Ultra, are designed for peripheral or point-of-care settings, and they detect MTBC DNA at low concentrations as well as common mutations conferring RIF resistance, but not INH resistance (3). The moderate-complexity automated NAAT (MC-aNAAT) platforms are designed for high-volume laboratories. They include the single-step FluoroType MTBDR and BD MAX MDR-TB, which simultaneously detect MTBC DNA and resistance-conferring mutations for INH and RIF, and the two-step reflex testing platforms Roche cobas MTB (with cobas MTB-RIF/INH) and Abbott RealTime MTB (with Abbott RealTime MTB RIF/INH), which first detect MTBC DNA and then detect resistance-conferring INH and RIF mutations (2, 3).

High analytical sensitivity in TB diagnostics requires multilocus targeting to effectively lower the limit of detection. This approach is used by leading LC-aNAAT systems, such as the Xpert MTB/RIF Ultra assay, which analyzes the multi-copy IS*6110* and IS*1081* insertion sequences (4), and by MC-aNAAT systems, such as the Roche cobas MTB assay, which targets 16S rDNA and esx genes (5, 6). Similarly, the Abbott RealTime MTB assay employs a dual-target strategy using IS*6110* and the PAB protein gene.

Until recently, the FluoroType MTBDR version 2 (FTv2old) lacked multilocus MTBC detection, resulting in lower sensitivity than other MC-aNAATs. To achieve comparable sensitivity, the manufacturer suggested using FTv2old as a reflex assay, in which MTBC-positive samples were first identified using FluoroType MTB (FT-MTB), a highly sensitive PCR, and then analyzed with FTv2old for resistance-conferring mutations (6, 7). The updated FluoroType MTBDR version 2 (FTv2new) now uses the multicopy IS*6110* insertion sequence as the primary amplification target to enhance the detection of *Mycobacterium tuberculosis* complex (MTBC) DNA. Thus, the primary goal of this upgrade is to bridge the previously observed sensitivity gap and improve MTB detection, particularly in paucibacillary specimens, while maintaining the high-throughput capacity of the FluoroCycler XT platform (8).

The aim of this study was to quantify the alleged increase in diagnostic sensitivity achieved by incorporating the IS*6110* target in FTv2new compared with FTv2old. This was assessed through a head-to-head evaluation with FTv2old using consecutive routine clinical specimens, with both assays performed on the same DNA extract.

## MATERIALS AND METHODS

### Study design and clinical specimens

This prospective diagnostic accuracy study was conducted at the International Reference Laboratory of Mycobacteriology from 29 April to 20 June 2024. Clinical specimens submitted for routine MTBC culture and PCR were included. According to the routine laboratory workflow, samples from patients without PCR testing in the preceding 12 months were analyzed, as were samples tested on request. The study included 1,483 specimens: 1,213 pulmonary and 270 extrapulmonary specimens (Table 1).

**TABLE 1:** Tested sample materials^*a*^

Disease category, *n*	Sample type, *n*	Sample group	Count, *n*	Contaminated, *n*	Valid, *n* (%)
Pulmonary (1,213)	Fluid (1,199)	Respiratory tract	681	12	669 (98)
		Sputum	474	39	435 (92)
		Gastric lavage	44	1	43 (98)
	Solid (14)	Lung and bronchial tissue	14		14 (100)
Extrapulmonary (270)	Fluids (175)	Serous fluid	85		85 (100)
		Feces	28	4	24 (86)
		Urine	19	5	14 (74)
		Pus	18	5	13 (72)
		Blood	15		15 (100)
		CSF	6		6 (100)
		Fluid (unspecified)	2		2 (100)
		Secretion	2		2 (100)
	Solid (95)	Tissue, unspecified	62	2	60 (97)
		Lymph node	26		26 (100)
		Bone	7		7 (100)
Total			1,483	68	1,415 (95)

^*a*^ The table count includes cultures that cannot be evaluated due to contamination.

### Sample processing and microscopy

Routine pulmonary and extrapulmonary specimens were analyzed by microscopy and PCR upon receipt, followed by culture on Löwenstein-Jensen (SSI Diagnostica, Hillerød, Denmark) and Mycobacterial Growth Indicator Tube (MGIT, Becton Dickinson, Sparks, MD) media. Specimens were decontaminated using the NALC-NaOH method (MycoPrep, Becton Dickinson) according to standard protocols, except specimens from normally sterile sites, such as cerebrospinal fluid, were processed without pretreatment (9). After processing, the sediments were used for culture inoculation, smear preparation for fluorescence microscopy, and PCR. Smears were stained with auramine–rhodamine and examined for acid-fast bacilli by fluorescent microscopy.

### Molecular testing and repeat protocol

DNA was manually extracted from each pretreated specimen using the FluoroLyse v1.0 kit (Bruker-Hain, Nehren, Germany). To ensure a direct comparison, the DNA extract was transferred to a separate tube, from which both the FluoroType MTBDR version 2 older (FTv2old) and the updated (FTv2new) were analyzed on the FluoroCycler XT instrument (Bruker-Hain) and automatically interpreted by the FluoroSoftware XT-IVD (Bruker-Hain). Only FTv2old results were reported for clinical management; FTv2new results were used solely for study purposes.

Repeat testing followed the laboratory’s routine protocol: if the FTv2old assay yielded an invalid (“INV”; inhibited) result or a positive result not supported by microscopy, both FTv2old and FTv2new were repeated using a new DNA extraction. However, due to workload constraints, no retesting was performed solely based on the FTv2new results. Indeterminate resistance results from both assays were compared against the reference standard without triggering repeat testing.

### Reference standard and resistance detection

Culture served as the primary reference standard for MTBC detection and was performed in liquid media using the Mycobacterial Growth Indicator Tube (MGIT; Becton Dickinson, Sparks, MD) and on solid Löwenstein-Jensen (LJ) medium (SSI Diagnostica, Hillerød, Denmark), with both incubated for up to 56 days. For comparison with PCR, culture positivity was defined as MTBC growth in a specimen obtained within 3 months. Cultures with no growth or growth of non-tuberculous mycobacteria (NTM) were classified as TB-negative. Resistance was determined by whole-genome sequencing (WGS) of MTBC isolates on the Illumina platform, with analysis conducted using the TB-Profiler pipeline (v3-6.0) (10). Phenotypic drug susceptibility testing was performed in MGIT when WGS identified mutations conferring resistance to first-line drugs.

### Statistical analysis

**Fig 1:**
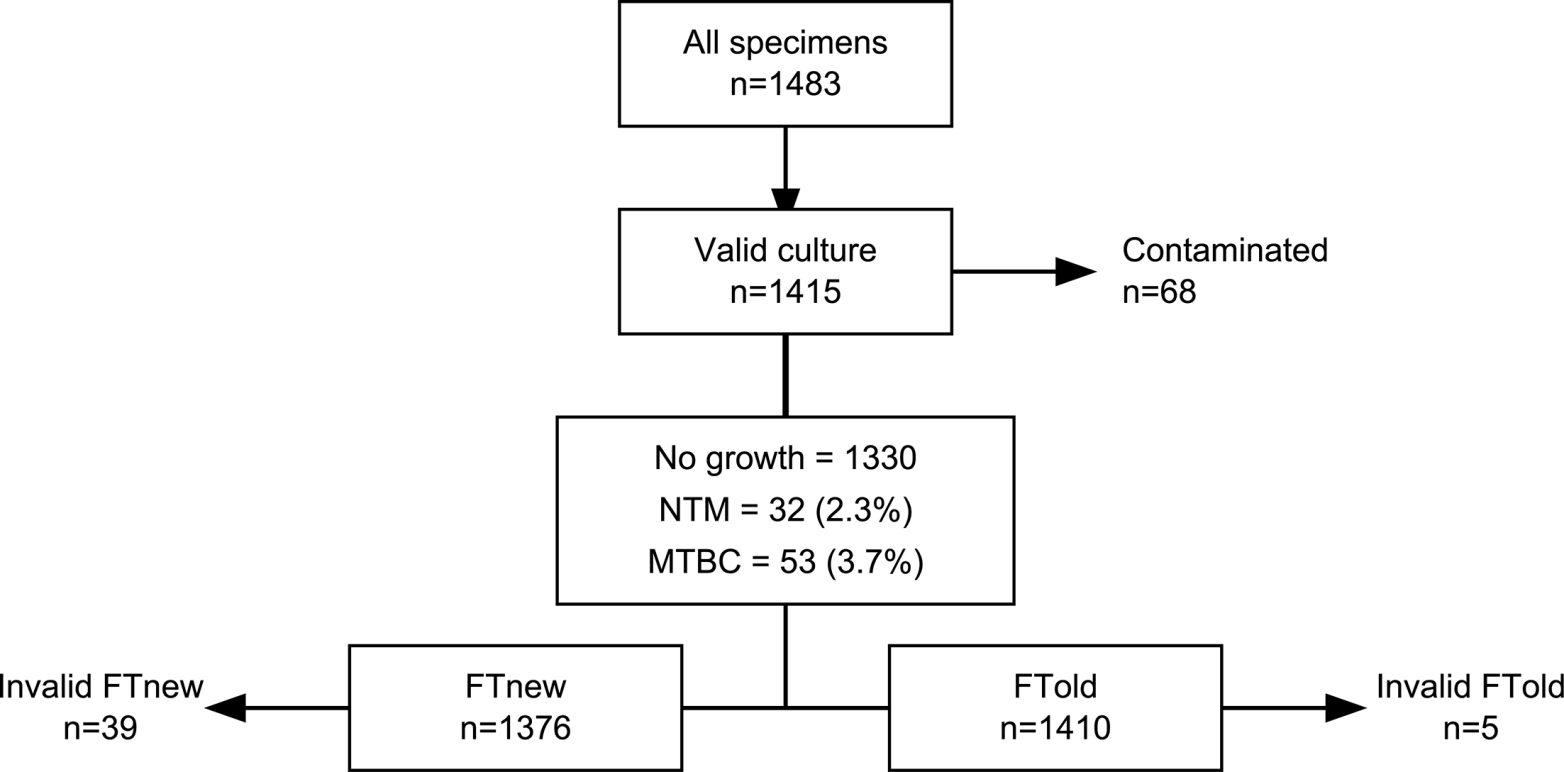
Flow diagram of the evaluation of FluoroType MTBDR v2 showing the processing of 1,483 specimens. After exclusion of 68 contaminated samples, 1,415 specimens yielded valid culture results. Of these, 53 were *Mycobacterium tuberculosis* complex (MTBC)-positive; the remaining MTBC culture-negative specimens included 32 non-tuberculous mycobacteria (NTM) and 1,330 specimens with no mycobacterial growth. The updated assay, FTv2new, generated 1,376 valid results, whereas FTv2old generated 1,410 valid results.

Diagnostic performance, including sensitivity and specificity, was calculated using culture as the reference standard. Samples with contaminated cultures were excluded from the final analysis (Fig. 1). Differences in sensitivity between assays were assessed using McNemar’s chi-square test with continuity correction, with *P* < 0.05 considered statistically significant. All analyses were performed in R (R Foundation for Statistical Computing).

## RESULTS

### Samples

We analyzed 1,483 consecutive clinical specimens, including 1,213 (81.8%) pulmonary and 270 (18.2%) extrapulmonary samples (Table 1). Pulmonary specimens comprised 474 sputum specimens and 739 other respiratory specimens, including bronchoalveolar lavage fluid and gastric lavage fluid. Extrapulmonary specimens consisted mainly of serous fluids (*n* = 85), tissues (*n* = 62), and lymph node aspirates (*n* = 26). After excluding 68 contaminated cultures (4.6%) (Fig. 1), we obtained 1,415 valid culture results, of which 53 (3.7%) were culture-positive for MTBC and 32 (2.3%) for NTM.

### Sensitivity and sensitivity gains

We observed a significant increase in diagnostic PCR yield with FTv2new. Nine MTBC cases were detected by FTv2new but missed by FTv2old, resulting in 46 true positives compared with 37 for FTv2old (Table 2). FTv2new demonstrated an overall sensitivity of 86.8% (95% CI: 75.2%–93.5%) against culture, whereas FTv2old showed a sensitivity of 69.8% (95% CI: 56.5%–80.5%) (Tables 2 and 3). This represents a statistically significant 17.0 percentage-point sensitivity gain (*P =* 0.008) (Table 3). Sensitivity was highest in sputum specimens, lower in other pulmonary specimens, and lowest in extrapulmonary specimens (Tables 2 and 3). Specificity remained high and comparable between assays, at 99.0% (95% CI: 98.3%–99.4%) for FTv2new and 99.1% (95% CI: 98.5%–99.5%) for FTv2old (Table 3).

**TABLE 2:** Results of primary PCR using FTv2new and FTv2old compared with culture results^*a*^

Sample group	TB culture, positives, *n*	FTv2new					FTv2old				
		Valid, *n*	TP	TN	FP	FN	Valid, *n*	TP	TN	FP	FN
Regardless of microscopy findings											
All	53	1,376	46	1,310	13	7	1,410	37	1,345	12	16
Sputum	38	429	35	385	6	3	432	31	390	4	7
Pulmonary TB, incl. sputum	47	1,139	42	1,084	8	5	1,157	35	1,102	8	12
Pulmonary, no sputum	9	710	7	699	2	2	725	4	712	4	5
Extrapulmonary	6	237	4	226	5	2	253	2	243	4	4
Microscopy-negatives											
All microscopy-negative	32	1,349	25	1,304	13	7	1,383	16	1,339	12	16
Sputum	19	406	16	381	6	3	409	12	386	4	7
Pulmonary, including sputum	27	1,113	22	1,078	8	5	1,131	15	1,096	8	12
Pulmonary, no sputum	8	707	6	697	2	2	722	3	710	4	5
Extrapulmonary	5	236	3	226	5	2	252	1	243	4	4

^*a*^ TP, true positive; TN, true negative; FP, false positive; FN, false negative.

**TABLE 3:** Differences in sensitivities and specificities of primary PCR using FTv2new and FTv2old compared with culture results, respectively

Sample type	FTv2new			FTv2old			Sensitivity difference, % (95% CI)	*P*-value^*a*^
	*n*	Sensitivity, % (95% CI^*b*^)	Specificity, % (95% CI)	*n*	Sensitivity, % (95% CI)	Specificity, % (95% CI)		
Regardless of microscopy findings								
All	1,376	86.8 (75.2–93.5)	99.0 (98.3–99.4)	1,410	69.8 (56.5–80.5)	99.1 (98.5–99.5)	17.0 (5.9–28.1)	0.008
Sputum	429	92.1 (79.2–97.3)	98.5 (96.7–99.3)	432	81.6 (66.6–90.8)	99.0 (97.4–99.6)	10.5 (0.2–20.8)	0.134
Pulmonary TB, incl. sputum	1,139	89.4 (77.4–95.4)	99.3 (98.6–99.6)	1,157	74.5 (60.5–84.7)	99.3 (98.6–99.6)	14.9 (3.9–25.9)	0.023
Pulmonary, no sputum	710	77.8 (45.3–93.7)	99.7 (99.0–99.9)	725	44.4 (18.9–73.3)	99.4 (98.6–99.8)	33.3 (−4.4 to 71.1)	0.248
Extrapulmonary	237	66.7 (30.0–90.3)	97.8 (95.0–99.1)	253	33.3 (09.7–70.0)	98.4 (95.9–99.4)	33.3 (−12.9 to 79.5)	0.480
Microscopy-negatives								
All microscopy-negative	1,349	78.1 (61.2–89.0)	99.0 (98.3–99.4)	1,383	50.0 (33.6–66.4)	99.1 (98.5–99.5)	28.1 (9.8–46.5)	0.008
Sputum	406	84.2 (62.4–94.5)	98.4 (96.7–99.3)	409	63.2 (41.0–80.9)	99.0 (97.4–99.6)	21.1 (0.4–41.7)	0.134
Pulmonary, incl. sputum	1,113	81.5 (63.3–91.8)	99.3 (98.6–99.6)	1,131	55.6 (37.3–72.4)	99.3 (98.6–99.6)	25.9 (6.7–45.1)	0.023
Pulmonary, no sputum	707	75.0 (40.9–92.9)	99.7 (99.0–99.9)	722	37.5 (13.7–69.4)	99.4 (98.6–99.8)	37.5 (−4.9 to 79.9)	0.248
Extrapulmonary	236	60.0 (23.1-88.2)	97.8 (95.0–99.1)	252	20.0 (03.6–62.4)	98.4 (95.9–99.4)	40.0 (−15.4 to 95.4)	0.480

^*a*^ Statistical significance was calculated using McNemar’s chi-square test with continuity correction.

^*b*^ CI, confidence interval.

The sensitivity gain was most pronounced in paucibacillary samples (Tables 2 and 3), to which all nine samples with increased true-positive counts belonged. Among microscopy-negative specimens (*n* = 1,349), we observed a 28.1 percentage-point increase in sensitivity, rising from 50.0% (95% CI: 33.6%–66.4%) with FTv2old to 78.1% (95% CI: 61.2%–89.0%) with FTv2new (*P =* 0.008). In pulmonary specimens, sensitivity increased from 74.5% to 89.4% (*P =* 0.023). In extrapulmonary specimens, we observed a substantial increase in sensitivity, from very low sensitivity (20%) with FTv2old to 60% in microscopy-negative specimens with FTv2new.

We identified a higher rate of invalid tests in the updated assay (Table 2; Fig. 1). Of the 1,483 samples tested, FTv2new produced 39 invalid results (2.6%), compared with five invalid results (0.3%) for FTv2old.

### Resistance detection

Concordance between RIF and INH resistance detection was evaluated in all 46 culture-positive specimens that yielded valid PCR results (Tables 4 and 5). FTv2new correctly identified all five RIF-resistant cultures, whereas FTv2old detected only four. One microscopy-negative specimen harboring the *rpoB* L430P mutation was correctly identified as RIF-resistant by FTv2new but misclassified as wild-type by FTv2old (WT; Table 4). However, FTv2new reported three samples as indeterminate (IND) for resistance, whereas FTv2old classified them as WT; all three were confirmed phenotypically susceptible by MGIT with no mutations by WGS.

**TABLE 4:** Rifampicin resistance target detection directly with FTv2new and FTv2old and resistance detection from culture with the phenotypic method and whole-genome sequencing^*d*^

Microscopy	Culture-based rifampicin resistance detection		Direct mutation detection		*n*
	MGIT	Mutation, WGS	FTv2new	FTv2old	
NEG	R	*rpoB* p.Leu430Pro	L430P	L430P	3
NEG	R	*rpoB* p.Leu430Pro	L430P	WT	1
NEG	S	WT^*a*^	IND	WT	2
NEG	S	WT	WT	WT	9
NEG	S	WT	WT	–^*b*^	2
NEG	S	WT	–	–	8
POS	R	*rpoB* p.Leu430Pro	L430P	L430P	1
POS	S	WT	IND^*c*^	WT	1
POS	S	WT	WT	WT	19
Total					46

^*a*^ WT, resistance mutation not detected.

^*b*^ –, target not detected.

^*c*^ IND, target detected, but indeterminate result.

^*d*^ MGIT, Mycobacterial Growth Indicator Tube (phenotypic DST); WGS, whole-genome sequencing; WT, wild-type; IND, indeterminate; R, resistant; S, susceptible; NEG, microscopy-negative; POS, microscopy-positive.

**TABLE 5:** Isoniazid resistance target detection directly with FTv2new and FTv2old and resistance detection from culture with the phenotypic method and whole-genome sequencing^*e*^

Microscopy	Culture-based isoniazid resistance detection		Direct mutation detection				*n*
	MGIT	Mutation, WGS	FTv2new *katG*	FTv2old katG	FTv2new *inhA*	FTv2old *inhA*	
NEG	R	*inhA* c.-154G>A	IND^*a*^	IND	WT^*b*^	WT	1
NEG	R	*katG* p.Ser315Thr	–^*d*^	–	–	–	1
NEG	R	*katG* p.Ser315Thr	IND	S315T1	WT	WT	1
NEG	R	*katG* p.Ser315Thr - *fabG1* c.-15C>T	S315T1	S315T1	c-777t^*c*^	c-777t	3
NEG	R	*katG* p.Ser315Thr - *fabG1* c.-15C>T	S315T1	WT	WT	WT	1
NEG	S	WT	IND	WT	WT	WT	4
NEG	S	WT	IND	–	IND	–	2
NEG	S	WT	WT	WT	WT	WT	4
NEG	S	WT	–	–	–	–	8
POS	R	*katG* p.Ser315Thr - *fabG1* c.-15C>T	S315T1	S315T1	c-777t	c-777t	1
POS	S	WT	IND	WT	WT	WT	1
POS	S	WT	WT	WT	WT	WT	19
Total	–		–	–	–	–	46

^*a*^ IND, target detected, but indeterminate result.

^*b*^ WT, no mutation detected.

^*c*^ The c-777t mutation is identical to *fabG1 *c.-15C>T (alternative nomenclature).

^*d*^ –, target not detected.

^*e*^ MGIT, Mycobacterial Growth Indicator Tube (phenotypic DST); WGS, whole-genome sequencing; WT, wild-type; IND, indeterminate; R, resistant; S, susceptible; NEG, microscopy-negative; POS, microscopy-positive.

Among eight INH-resistant isolates (Table 5), the highest concordance between culture-based WGS and testing directly from the clinical specimen was observed for the *katG* p.Ser315Thr plus *fabG1* c.-15C>T genotype. Both FTv2new and FTv2old detected resistance mutations in four isolates. The remaining four isolates yielded indeterminate results or failed to detect the target. Among 38 culture-susceptible isolates, neither assay detected resistance mutations, with results reported as WT (*n* = 23) or missing target (*n* = 8). The remaining eight culture-susceptible specimens lacked resistance mutations but had incomplete FT results and were, therefore, not comparable.

## DISCUSSION

The principal finding of this study is that including the multicopy IS*6110* insertion sequence target in the FTv2new assay significantly increases diagnostic yield by identifying nine additional PCR-positive samples, all of which were microscopy-negative. Overall sensitivity increased by 17 percentage-points, and the sensitivity of microscopy-negative specimens increased by 28 percentage-points. This substantial gain mirrors the performance improvement observed when Xpert MTB/RIF was replaced by Xpert MTB/RIF Ultra, which incorporated multi-copy targets (IS*6110* and IS*1081*) and achieved a sensitivity of 80%, corresponding to an increase of up to 17 percentage-points in smear-negative sputum samples compared with its predecessor (4, 11). These findings underscore that targeting multi-copy genomic regions is essential to overcome the sensitivity limitations of earlier versions of FluoroType MTBDR.

FTv2old relied on a single MTBC target (*rpoB*) for MTBC detection, resulting in lower analytical sensitivity than other MC-aNAAT platforms, such as Abbott RealTime MTB, BD MAX MDR-TB, and Roche cobas MTB, which employ multitarget strategies (6). For example, the cobas MTB assay on sputum has demonstrated 82% sensitivity in smear-negative sputum samples (5), while the BD MAX MDR-TB assay reported 81% sensitivity in smear-negative respiratory specimens (12). The Abbott RealTime MTB assay has shown 66% sensitivity in smear-negative sputum samples (13). As shown in this study and our previous study (8), FTv2old and earlier versions of FluoroType MTBDR showed lower sensitivity (63%) than other MC-aNAATs because they were primarily designed as reflex tests following a positive FT-MTB screen, which targets IS*6110* and *rpoB* for MTBC detection. By incorporating IS*6110* detection directly into the FT-MTBDR assay, FTv2new effectively bridges this performance gap and eliminates the need for a multistep reflex workflow. This advancement is particularly significant in clinical settings where laboratories, like ours, bypassed the initial FT-MTB screening assay despite the manufacturer’s recommendation, prioritizing a rapid response that includes simultaneous resistance profiling. For laboratories that previously relied on the highly sensitive FT-MTB for primary detection and FTv2old for resistance profiling, the workflow is now considerably streamlined. Accordingly, based on literature comparisons, FTv2new demonstrates sensitivities comparable to other multitarget molecular diagnostics, including Xpert MTB/RIF Ultra (11) and high-throughput systems such as Roche cobas MTB, Abbott RealTime MTB, and BD-MAX MDR-TB (5, 13–17). These literature-based comparisons should be interpreted cautiously due to differences in study populations and methodologies. As for the other MC-aNAATs and LC-aNAATs, the sensitivities for detecting the resistance targets are lower because those are single-locus. When a specimen is positive for MTBC but lacks results for resistance targets, the treatment should follow the national or local tuberculosis program.

The increased sensitivity is particularly relevant for analyzing paucibacillary specimens, including many extrapulmonary samples. In extrapulmonary, microscopy-negative TB specimens, FTv2new achieved a sensitivity of 60%, representing a 40-percentage-point improvement over FTv2old. These numbers should be interpreted with some caution due to the very small number of culture-positive specimens; however, the increase in the detection rate is in the same DNA extracts. Although published data on MC-aNAATs’ performance in extrapulmonary specimens remain limited and vary considerably by the specimen type, FTv2new appears to perform comparably to other MC-aNAATs, such as the BD MAX MDR-TB assay, which reported an overall sensitivity of 52% (18), and the Abbott RealTime MTB, which reported a sensitivity of 76% in smear-negative extrapulmonary samples (13). Furthermore, the 60% sensitivity observed for FTv2new is broadly within the range reported for Xpert MTB/RIF Ultra in extrapulmonary specimens, which vary widely by the specific specimen type (19).

The FTv2new had a higher invalid rate (2.6%; 39/1,483) than FTv2old (0.3%; 5/1,483). We believe this difference is primarily attributable to operational factors: FTv2new testing was performed once daily in large end-of-day batches, which increased the risk of handling errors, whereas FTv2old testing was conducted in smaller batches three times daily. As under routine operations, an invalid FTv2old result triggered a repeat test, but an invalid FTv2new result did not trigger a repeat test unless the corresponding FTv2old result was also invalid. In routine operations, invalid FTv2new results would also be repeated, likely lowering the final rate.

With improved sensitivity for MTBC detection, FTv2new aligns more closely with current WHO recommendations for MC-aNAATs than FTv2old. The WHO Consolidated Guidelines recommend that rapid NAATs be used as the initial diagnostic test for all individuals with suspected TB to enable early detection and drug susceptibility assessment (2). FTv2new identifies resistance-conferring mutations, as do Abbott RealTime MTB, BD MAX MTB-RIF/INH, and cobas Roche MTB, but it uniquely provides details on the specific mutations detected. In this study, only a limited number of resistant isolates were identified, consistent with Denmark’s low TB incidence and low prevalence of drug resistance. FTv2new detected all five RIF-resistant isolates, including a L430P *rpoB* mutation missed by FTv2old; however, it also produced more indeterminate resistance results. Nearly all resistant samples (except one) were microscopy-negative, suggesting low DNA concentrations, which likely contributed to missed or indeterminate results in both assay versions. Increased sensitivity of MTBC detection by adding IS*6110* does not translate into increased sensitivity of resistance mutation target detection because the targets, like in the other MC-aNAATs, are single-copy. Initial treatment of patients with MTBC-positive-only results would require empirical therapy according to TB programs. WGS confirmed all resistance mutations detected by either assay. Given the low incidence of resistant TB cases in Denmark, conclusions about FTv2new resistance detection should be interpreted cautiously.

A major strength of this study is its prospective head-to-head design and a large cohort of 1,483 consecutive clinical specimens. Parallel testing of the same DNA extracts minimized preanalytical bias and enabled direct comparison of assay performance. Using WGS for resistance determination further facilitated comparison of the interpretation of resistance targets in the primary PCR. A limitation of the study is that FTv2new testing could be performed only at the end of the workday, often in large batches, which may have contributed to the higher invalid rate observed with FTv2new. Repeat testing of FTv2new-only invalid results was not feasible because of workload and logistical constraints. The study cannot be repeated as FTv2old is no longer manufactured; the comparison was possible only during a brief transition period when both assay versions were available.

In conclusion, the updated FluoroType MTBDR v2 (FTv2new) assay demonstrates significantly improved sensitivity for MTBC detection compared with earlier versions, eliminating the need for reflex testing to achieve higher analytical sensitivity. Its performance appears comparable to that of other MC-aNAAT systems. Although resistance detection remains unchanged across versions, FluoroType MTBDR retains the ability to specify individual resistance mutations, a feature not shared by other MC-aNAATs.

## Data Availability

Whole-genome sequencing (WGS) was performed as part of routine diagnostic resistance characterization in place of phenotypic testing. In this study, WGS served primarily to confirm mutations detected by the assays. Relevant resistance-associated variant calls, where present, were included with the isolate metadata in ENA project PRJEB112707.
